# Economic analysis of adaptive strategies for flood risk management under climate change

**DOI:** 10.1007/s11027-015-9637-0

**Published:** 2015-03-01

**Authors:** Thomas D. van der Pol, Ekko C. van Ierland, Silke Gabbert

**Affiliations:** Environmental Economics and Natural Resources Group, Wageningen University, Hollandseweg 1, 6706 KN Wageningen, The Netherlands

**Keywords:** Flood risk management, Cost-benefit analysis, Climate change, Flexibility, Learning, Robustness

## Abstract

Climate change requires reconsideration of flood risk management strategies. Cost-benefit analysis (CBA), an economic decision-support tool, has been widely applied to assess these strategies. This paper aims to describe and discuss probabilistic extensions of CBA to identify welfare-maximising flood risk management strategies under climate change. First, uncertainty about the changes in return periods of hydro-meteorological extremes is introduced by probability-weighted climate scenarios. Second, the analysis is extended by learning about climate change impacts. Learning occurs upon the probabilistic arrival of information. We distinguish between learning from scientific progress, from statistical evidence and from flood disasters. These probabilistic extensions can be used to analyse and compare the economic efficiency and flexibility of flood risk management strategies under climate change. We offer a critical discussion of the scope of such extensions and options for increasing flexibility. We find that uncertainty reduction from scientific progress may reduce initial investments, while other types of learning may increase initial investments. This requires analysing effects of different types of learning. We also find that probabilistic information about climate change impacts and learning is imprecise. We conclude that risk-based CBA with learning improves the flexibility of flood risk management strategies under climate change. However, CBA provides subjective estimates of expected outcomes and reflects different decision-maker preferences than those captured in robustness analyses. We therefore advocate robustness analysis in addition to, or combined with, cost-benefit analysis to support local investment decisions on flood risk reduction and global strategies on allocation of adaptation funds for flood risk management.

## Introduction

Cost-benefit analysis (CBA) has been widely applied to flood risk management strategies, and its application has become more complex due to climate change (van Dantzig 1956; Zhu and Lund 2009; Kind 2014; Lickley et al. 2015). This paper discusses probabilistic extensions of CBA to identify welfare-maximising strategies under climate change. Flood risk, here defined as the expected monetary loss from floods, is increasing in many regions of the world due to population growth, economic growth and urbanisation, and due to the impacts of climate change on weather patterns, peak river discharges and sea levels (Milly et al. 2002; Groisman et al. 2005; Jongman et al. 2012). Flood risk is composed of the product of flood hazard, flood exposure and vulnerability (Kron 2005; IPCC 2014). To mitigate flood risk locally, flood risk management strategies can be implemented that either mitigate flood hazard, exposure or vulnerability. Flood hazard mitigation is achieved by implementing flood protection measures over time which lower flood frequencies in vulnerable areas. Examples are restoration of sand dunes and beach nourishment in coastal areas, raising or relocating dikes in river basins and extension of urban drainage capacities in urbanised areas. The harmful consequences of flooding are reduced by flood exposure mitigation through spatial planning and by flood vulnerability mitigation, for example, by implementing building codes and preparation of emergency plans (Hooijer et al. 2004; Wu et al. 2012; Stive et al. 2013; Hanley et al. 2014).

Flood protection measures, especially engineering-based measures, will continue to place a significant burden on national budgets, and this trend is reinforced by climate change (Narain et al. 2011). Due to climate change, distributions of weather extremes, peak river discharges and water levels can no longer be assumed to be statistically stationary (Milly et al. 2008). Moreover, flood protection measures typically have long technical lifetimes, and their protection levels are highly sensitive to climate change (Gersonius et al. 2013). It is therefore important to identify economically efficient flood risk management strategies, i.e. welfare-maximising investments in flood protection measures and other flood risk reducing measures over time, in response to current changes in climate and in anticipation of future climate change.

Economic analysis of flood risk management strategies aims to efficiently reduce the frequency and the consequences of various flooding events. These include low-probability high-consequence flooding events, typically coastal and fluvial floods and high probability-low consequence flooding events, for example, flash floods in urbanised areas (Vrijling 2001; Wu et al. 2012). The latter may cause damages if storage and drainage capacities are insufficient to detain, retain or convey storm water from heavy rainfall. From the perspective of a risk-neutral social planner, net present value (NPV) estimates of the total expected damage costs from floods and the costs of protection determine the relative importance of investing in flood risk reduction.

The objective of CBA is to maximise the stream of discounted net benefits or to evaluate whether or not an investment project improves social welfare (Boardman et al. 2011). CBA compares avoided damages of different flood risk management strategies, the monetised benefits of flood risk reduction, to their costs (Jonkman et al. 2008; Zhu and Lund 2009; Dierauer et al. 2012). It has, however, remained challenging to include climate change uncertainties in CBA of flood risk management strategies. There is, amongst others, hydrologic uncertainty about the effects of climate change on weather extremes, peak river discharges and sea levels, and about the possible emergence of new information about these changes over time. Furthermore, the outcomes of an economic analysis of flood risk management strategies are sensitive to a range of other uncertainties, including those originating from hydraulic, structural and economic uncertainties (Bao et al. 1987).

This paper restricts attention to the impacts of climate change on hydrologic uncertainty. Hydrologic uncertainty manifests, for example, through model uncertainty about the type of a peak flow distribution, and statistical uncertainty about its parameters over time. Moreover, hydrologic uncertainty is partly epistemic, which, in contrast to natural or inherent uncertainty, can be reduced or resolved by acquiring more knowledge (Merz and Thieken 2005).

The need to anticipate the possible emergence of new hydrologic information and to identify adaptive flood risk management strategies under climate change uncertainty is increasingly recognised; both in risk-based economic optimisation approaches, as well as in recently developed robustness approaches (Kwadijk et al. 2010; Woodward et al. 2011; Gersonius et al. 2013; Haasnoot et al. 2013). However, until now, scientific consensus is lacking both on how to address climate change uncertainty and on how to incorporate learning in economic analysis of flood risk management and other climate change adaptation strategies (Watkiss et al. 2014). Moreover, probabilistic models to analyse efficient flood risk management strategies with new information are relatively scarce, and usually consider only one type of new information (Woodward et al. 2011).

The central research questions of this paper are as follows:

(i) How can probabilistic extensions of CBA using climate and learning scenarios be applied to improve decision-making on flood risk management?
(ii) What are advantages and limitations of such probabilistic extensions?

These questions are relevant to inform flood adaptation decisions at different scales, from local decisions on flood risk management strategies to global decisions on, for example, the allocation of adaptation funds. This paper therefore informs adaptation strategies at different scales through economic analysis to mitigate flood risk under climate change.

In this paper, two types of probabilistic extensions of CBA are considered. First, uncertainty about the changes in return periods of hydro-meteorological extremes is introduced by probability-weighted climate scenarios. Second, CBA is extended by probabilistic arrival of new information, hereafter called learning, about climate change impacts to introduce two phenomena: (i) the reduction of epistemic uncertainty and (ii) the arrival of new data. We elaborate on different types of learning, from scientific progress, from statistical evidence, or from flood disasters, and discuss their reinforcing or opposite effects on optimal investment. The methods to implement these types of learning originate from statistics and the real options literature and have been widely used in many domains (Copeland and Antikarov 2003; Press 2003). We describe their generic implementation in a non-technical manner, and discuss the availability of the required probabilistic information and implications for the economic efficiency and flexibility of flood risk management strategies under climate change. Finally, we contrast cost-benefit approaches with robustness approaches, which follow a different line of analysis. We discuss the general findings and provide suggestions for the use of methods to support decisions on flood risk management strategies.

## Climate change uncertainty and the climate change learning process

### Current approaches for including climate change uncertainty in flood risk management decisions

Frequency analysis of extremes is required to estimate future flood risk but is plagued by hydrologic uncertainty. In the literature, a variety of methods have been developed to assess return periods of extreme hydro-meteorological events under climate change. Examples are perturbation methods used to specify design rules or design storms with climate model simulations, and regression methods to remove effects of serial dependence and non-stationarity in hydro-meteorological observations (Khaliq et al. 2006; Willems 2013). Until now, however, it has remained unclear how the performance of flood defences and urban drainage systems should be assessed under climate change (Kundzewicz et al. 2010; Berggren et al. 2014). One approach is to exclude non-stationarity in the flood hydrology from the economic analysis of flood risk management strategies, which is still occasionally observed in theoretical work (Zhu and Lund 2009). However, in the adaptation literature, it has been emphasised that anticipatory adaptation is required to support efficient decision-making on investments with fixed costs and long technical lifetimes (Smith 1997). Here, we provide a brief description of two important methods for water system design with the anticipation of climate change impacts; (i) a fixed factor increase of design intensities, for example, derived from the so-called delta-change method, for urban drainage design (Hay et al. 2000; Arnbjerg-Nielsen 2012), and (ii) the use of a prior distribution, for example, of sea level rise, to estimate future coastal flood risk (Purvis et al. 2008).

Rainfall inputs used to evaluate the performance of urban drainage systems are design storms, either from frequency analysis of annual maxima, or partial duration series, or are obtained from continuous simulation (Cameron et al. 1999; Boughton and Droop 2003; Cameron 2006; Mailhot et al. 2013). With the design storm method, a rainfall depth of an assigned duration with a given return period is applied to a site (De Michele et al. 1998). This method assumes that the average return period of the design storm coincides with the average return period of a flow rate, if an appropriate duration is selected, together with one or more representative synthetic storm hyetographs (Levy and McCuen 1999; Mays 2011). To account for climate change, the design storm can be increased with a fixed factor (Waters et al. 2003). The appropriate uplift factor for the intensity of the design storm can be chosen pragmatically, or derived from the delta-change method, where rainfall events in a historical rainfall series are increased with delta-change factors. These factors, which are different for different rainfall intensities, are derived from the output of a regional climate model (Nilsen et al. 2011; Arnbjerg-Nielsen 2012). Uplift factors are, however, usually obtained per climate change scenario. Such studies usually do not provide estimates of total expected discounted costs of flood risk management strategies under multiple climate futures provided that a certain uplift factor is chosen for the design of flood protection measures.

Global climate change projections have remained highly uncertain. As a consequence, most projections are presented without probability distributions (Andronova and Schlesinger 2001; IPCC 2014). Prior distributions about regional climate change impacts are required to estimate the expected damage reduction over time associated with flood risk management strategies. It is, however, questionable whether or not such expected damage estimates can be obtained in the first place. Weitzman (2009), for example, argued that it may be inappropriate to perform CBA under uncertain fat-tailed extreme value distributions containing low-probability but catastrophic events.

Nonetheless, some attempts have been made to estimate future flood risk with probabilistic methods. Purvis et al. (2008) defined a triangular distribution for sea level rise where probabilities are set with IPCC scenarios based on the best estimate and the lower and upper bound of the scenarios. This distribution, however, does not account for low-probability climate change scenarios, which, in expected terms, may be important to determine optimal flood risk management strategies. Van der Pol et al. (2014) showed this for a normal and log-normal distribution for dike investments. Flood risk uncertainty is even larger if socioeconomic uncertainties are considered as well. Hall et al. (2005), for example, studied flood risk under climate change and socioeconomic scenarios. Furthermore, Bouwer et al. (2010) constructed loss-probability curves for a range of climate and socioeconomic scenarios under a range of flood scenarios.

### Expert elicitation

Water defence systems are typically designed to withstand plausible high-end scenarios of flood-related frequency changes, which would require different flood protection measures and larger investments than under less severe climate change impacts (Katsman et al. 2011). Estimates of the likelihood of more extreme climate scenarios are therefore crucial to study the economic efficiency of flood risk management strategies with risk-based economic optimisation approaches using probability-weighted climate scenarios. In the previous section, we discussed that only few studies examining the economic efficiency of flood risk management strategies have applied prior probability distributions about climate change impacts. In addition, the design of many urban flood protection measures has been based on a single climate change scenario. Such pragmatic approaches do not seem satisfactory, as expected damage costs may greatly increase if the incidence of extreme weather events or high water levels is underestimated. This raises the question of whether or not it would be more appropriate to obtain prior distributions about climate change impacts from expert elicitation.

One could, for example, think of the Delphi method as a means to obtain priors from expert elicitation (Dalkey and Helmer 1963). Many climate experts reject participation in Delphi surveys and expectations of participants are too diverse to reach consensus and to arrive at a single prior distribution specifying likelihoods of rapid climate change (Arnell et al. 2005). Existing studies reveal that some high-end scenarios, for example, the collapse of the West-Antarctic ice sheet, are considered to be unlikely in the near term (Vaughan and Spouge 2002). Satellite observations confirm this finding, although there is now strong evidence for partial ice-sheet thinning (Vaughan 2008). Despite the controversy of expert elicitation, it might provide valuable insights on the physical processes that are more or less likely to happen, which can be used to derive likelihood statements about climate change effects on, for example, the weakening of the Atlantic meridional overturning circulation (Zickfeld et al. 2007). Expert opinion can also be used for, for example, defining climate model parameter ranges for perturbation purposes or to distinguish between climate models based on quality (Stainforth et al. 2005; Knutti et al. 2010).

From the above, we draw the following conclusions. First, decision-making on flood risk management strategies under climate change cannot be classified as decision-making under risk. This is because the probabilistic changes in the frequency of occurrence of the relevant hydro-meteorological extremes under climate change are not well defined. Second, it can also not be classified as decision-making under ignorance, as there appears to be at least some consensus that some scenarios are less likely than others. This case is therefore best described as decision-making under uncertainty characterised by imprecise probabilities (Hogarth and Kunreuther 1995).

### Learning about the impacts of climate change

In addition to uncertainty about climate change impacts, there is uncertainty about the detection of future climate change signals, and uncertainty about how decision-makers’ investment decisions may respond to possible climate signals. In this section, we discuss different types of learning and their effects on climate change adaptation decisions. We distinguish between learning from scientific progress, from statistical evidence, and from flood disasters.

#### Information from scientific progress

Several authors have argued that climate change uncertainty cannot be expected to reduce or to be resolved any time soon. Leach (2007), for example, concluded that it may take hundreds if not thousands of years before reliable parameter estimates of climate models will become available. According to Roe and Baker (2007) uncertainty about climate change projections has not significantly decreased over the past decades, and showed, furthermore, that the probability of large temperature increases is relatively insensitive to reductions in climate change process uncertainties.

Information from scientific progress, in contrast, might reduce uncertainty about climate change impacts. Scientific progress can be modelled as probabilistic events of information. Uncertainty reduction introduces a trade-off between costs associated with immediate action, and increased exposure resulting from a learn-than-act strategy. The conditions required for immediate action to reduce emissions of a harmful pollutant such as CO_2_ have been analysed by Gollier et al. (2000), in which the degree of risk-aversion, irreversibility and the information rate determines optimal action. Ingham et al. (2007) presented a model with both mitigation and adaptation, and showed that the possibility to adapt together with the prospect to learn tends to reduce climate change action today. In earlier work, Kolstad (1994) studied mitigation and adaptation decisions under perfect learning, and investigated trade-offs between the effects of irreversibilities associated with the accumulation of greenhouse gases, and the accumulation of abatement capital.

If uncertainty is epistemic, and expected to gradually reduce over time, flexible adaptation measures may be more economically efficient than inflexible measures. Flexible climate change adaptation with learning from scientific progress can be studied with risk-based optimisation approaches, such as real options analysis, as well as robustness approaches (Copeland and Antikarov 2003; Hallegatte et al. 2012). De Bruin and Ansink (2011), for example, distinguished between structural measures with relatively high fixed costs, such as dikes, and non-structural measures with relatively low fixed costs, such as beach nourishment. For analysis of an adaptation measure in isolation, the expected value of information is compared with the additional costs from immediate investment in the measure. Hence, future learning through scientific progress may have impacts on investment timing, size and portfolio of flood risk management measures, and may also have an impact on greenhouse gas mitigation strategies. Assumptions about the probabilities of learning in the near future and the degree of uncertainty reduction are, however, determinants of initial investment strategies and the optimal responses to new information over time (van der Pol et al. 2014).

#### New hydro-meteorological observations: evidence-based learning

New hydro-meteorological observations are an important source of new information on changing flood risk. New hydro-meteorological observations may provide statistical evidence regarding climate change impacts on flood regimes in the future. It has, however, remained difficult to statistically detect changes in weather patterns, river flows and acceleration of mean sea level rise. Short-term trend detection is, amongst others, difficult due to multi-annual serial correlation, variability, and sample size. Fowler and Wilby (2010) reported that the detection of changes in seasonal precipitation may take several decades, and that this could motivate a precautionary approach to climate change adaptation. Zhang et al. (2004), furthermore, compared detection methods by simulations of 50 and 100 years of annual maxima. The simulation results show, in addition to differences in the ability to detect trends, that none of the methods guarantee trend detection, and that the probability of no-detection is much lower for larger sample sizes for all methods. Hamed (2008) showed that significant river flow trends found in earlier annual flow maxima may be false if scaling is considered. Wahl et al. (2013) reported that the rate of sea level rise over the past two to three decades has been high as compared to the long-run average, but that similar periods of high sea level rise have been observed at other times.

New hydro-meteorological observations can also be applied to evaluate water system performance over time. Because new weather and water level observations are not likely to reveal much information on structural climate change impacts in the near future, one might be tempted to think that it is irrelevant to consider the likelihood and effects of future observations on current decisions on flood protection measures. This is, however, a misconception. Weather variability and climate change uncertainty result in variability of best estimates of flood probability, which, in turn, results in uncertainty about the timing of new investments in flood protection measures, for example, through the need to meet a flood protection standard. However, evidence-based flood probability evaluation over time, and its effects on the economic efficiency of flood risk management strategies, has largely remained unexplored in flood risk management practises. It might, however, be highly relevant for efficient decision-making. First, in contrast to learning from scientific progress, it is certain that new data will become available over time. Second, despite white noise, extreme value data may still be applied to analyse changes in flood risk and evaluate water system performance in the future. The underlying statistical beliefs, however, may deviate from actual frequency distributions. Whereas learning from scientific progress is about anticipation of better information by uncertainty reduction, learning from statistical evidence is about anticipation of new information that is not necessarily better. Effects of white noise can be opposite to the effect of uncertainty reduction on optimal investment; underestimation of a system’s performance might trigger new investments, which can be anticipated by enlarging initial investments, while learning from scientific progress tends to reduce overall investment until better information becomes available. We will explain the latter in more detail in Section 3.3.1.

#### Disasters: incident-based learning

Historic flood observations show that large-scale flooding events often lead to large-scale investments in flood protection measures in developed countries that go far beyond repair. Examples that lead to such large-scale investments include the 1953 flood in the Netherlands, the New Orleans flood by hurricane Katrina and the flood by the 2011 tsunami in Japan. There are several explanations why investments in flood protection measures often take place after large-scale incidents, but the phenomenon remains intriguing. Clearly, a failure of a flood defence shows that a specific load has been greater than the resistance of the defence. In a statistical sense, however, any single observation has only a modest impact on extreme value estimates, even if its value is several times larger than ever measured before (Coles and Pericchi 2003).

In some cases, an incident may reveal new failure modes of, for example, a dike, and the flood probability estimate can be updated with this new information. This, however, does not explain large-scale investments for cases where a conventional failure mode, for example overtopping, was the primary cause of flooding. Neumayer et al. (2014) argued that both individuals and governments have incentives to underinvest in flood control. While this would explain large-scale re-investments after a disaster for cases with a maintenance backlog, it provides insufficient explanation for cases without backlog. Another motivation for re-investment is the “never again”-argument, often heard after large-scale disasters (Gerritsen 2005). A possibly related argument is victim pressure, where victims may have a disproportionate share in the decision process on flood control (Harries and Penning-Rowsell 2011).

Perhaps it is not important to understand why re-investment takes place after a disaster, as long as the likelihood of incidents occurring earlier than expected, together with the following investment response, are included in the economic analysis of flood risk management strategies. To illustrate this, consider that a decision-maker can either invest in flood protection measure 1, with total expected discounted costs A, consisting of both investment costs and total expected discounted damage costs, or in measure 2, with total costs B, consisting of investment costs only and no expected damage costs. Furthermore, consider that A < B. At first sight, a risk-neutral decision-maker would prefer measure 1. As an extension, consider that the public will demand to invest in never-again measure 2 if a disaster happens, and that the decision-maker knows this in advance. Consider, furthermore, that the probability that a disaster happens during the technical lifetime of flood protection measure 1 is larger than zero. Now, despite that the decision-maker is assumed to be risk-neutral, the risk-attitude of the public leads to a change in the expected economic efficiency of measure 1 through the probability of disaster during the lifetime of the infrastructure. As a result, measure 2 might be preferred.

## Economic models for flood risk management

In this section, we turn to economic models for analysing and comparing flood risk management strategies. We describe how a cost-benefit model can be extended with climate scenarios to include uncertainty about climate change impacts. We also provide a brief introduction to the modelling of the different types of learning that were introduced in the previous sections (2.3.1–2.3.3).

### Cost-benefit optimisation

Benefits of flood risk management can be estimated in monetary terms by estimating the expected reduction in damages. Flood damage models can be used for this purpose (de Moel and Aerts 2011). An optimal investment strategy can be obtained by balancing discounted expected damages and costs of protection. In early work, van Dantzig (1956) applied this concept to determine optimal dike height. Improved versions of this model are still used today in the Netherlands to analyse optimal dike height strategies and to determine economically efficient flood protection standards (Brekelmans et al. 2012; Kind 2014). It is, furthermore, increasingly recognised that, especially in a context of climate change uncertainty, different types of flood protection measures have to be considered simultaneously (de Bruin and Ansink 2011; Woodward et al. 2011; Meyer et al. 2012).

Many urban drainage system elements have been designed with particular design storms and simple flow calculations instead of cost minimisation models, and only few storm water models include an economic analysis of alternative storm water management strategies (Zoppou 2001). Moreover, detailed CBA studies of urban drainage systems are rare (Pathirana et al. 2011). One possible explanation could be that in many countries CBA may not be legally required for such systems. In the Netherlands, for example, uniform flood protection standards have been defined for regional water systems per land use type, rather than setting flood protection standards at welfare-maximising levels for every water system (NBW 2005; Hoes and Schuurmans 2006). Cost-effectiveness approaches are, hence, still predominant for urban drainage system design. However, if flood protection standards are set at economically efficient levels, the solutions of cost-benefit analysis and cost-effectiveness analysis with an efficient flood protection standard will coincide. Therefore, in what follows, we continue with a cost-benefit model only.

In a CBA, the objective of a risk-neutral decision-maker is to maximise the net present value of the total expected net benefits associated with a portfolio of flood protection measures over time (Eq. 1):

1 $$ W=\underset{z_{i,t}}{ \max }E\left\{{\displaystyle \underset{0}{\overset{T}{\int }}}\left({B}_t\left({x}_{1,t},{x}_{2,t},\dots \right)-{C}_t\left({z}_{1,t},{z}_{2,t},\dots \right)\right){e}^{-\delta t}dt\right\} $$

where *z*_*i*,*t*_ is the decision variable, and *x*_*i*,*t*_ is the stock of the flood protection measure *m*_*i*_ at system node *i* = 1, 2, …. System nodes, for example, can be segments of a dike ring, open channels of a surface system, or pipe segments of a sewer system. *B*_*t*_ is a benefit function, *C*_*t*_ is a protection cost function, *δ* is the discount rate, and *T* represents the end of the considered time horizon. Benefits of flood risk management can be modelled as reduced damages, but expected damages from floods can also be interpreted as costs. By symmetry, minimisation of total expected discounted loss *L* yields the same optimal investment strategy as under Eq. (1):

2 $$ L=\underset{z_{i,t}}{ \min }E\left\{{\displaystyle \underset{0}{\overset{T}{\int }}}\left({D}_t\left({x}_{1,t},{x}_{2,t},\dots \right)+{C}_t\left({z}_{1,t},{z}_{2,t},\dots \right)\right){e}^{-\delta t}dt\right\} $$

**Fig. 1:**
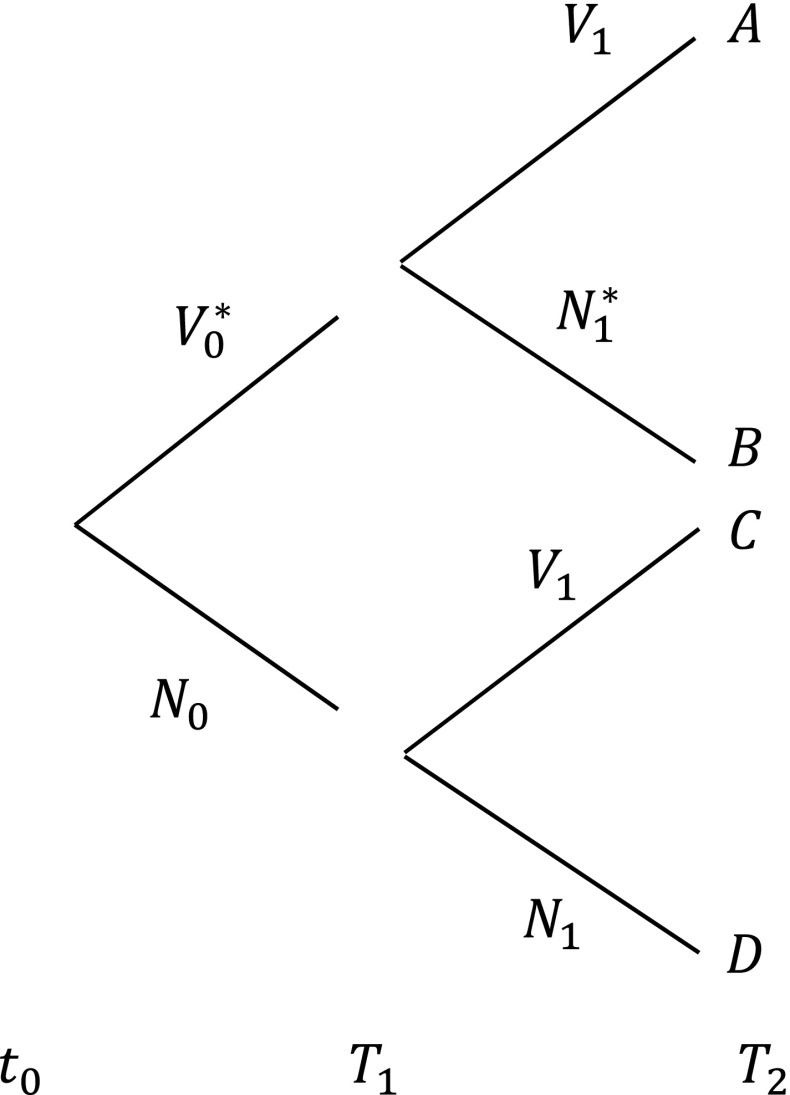
Example of a two-period decision tree with decisions to invest (*V*_0_; *V*_1_) or not invest (*N*_0_; *N*_1_ at *t* = *t*_0_ and at *t* = *T*_1_, respectively

Consider a two-period model with a binary decision to invest (*V*_0_) or not invest (*N*_0_) at *t*_0_, followed by the binary decision to invest (*V*_1_) or not invest (*N*_1_) at *t* = *T*_1_. This is displayed in Fig. 1.

The optimal strategy follows from outcome minimisation, i.e.: min{*A*, *B*, *C*, *D*}, and results in investment strategy (*V*_0_^*^, *N*_1_^*^) in this example. This example represents a deterministic CBA, as the outcomes of different strategies are assumed to be known with certainty. However, a deterministic CBA is not suitable under climate change uncertainty, as multiple climate futures are possible (Watkiss et al. 2014). Therefore, we now turn to a probabilistic extension of CBA using probability-weighted climate scenarios.

### Probabilistic modelling of climate change impact scenarios

**Fig. 2:**
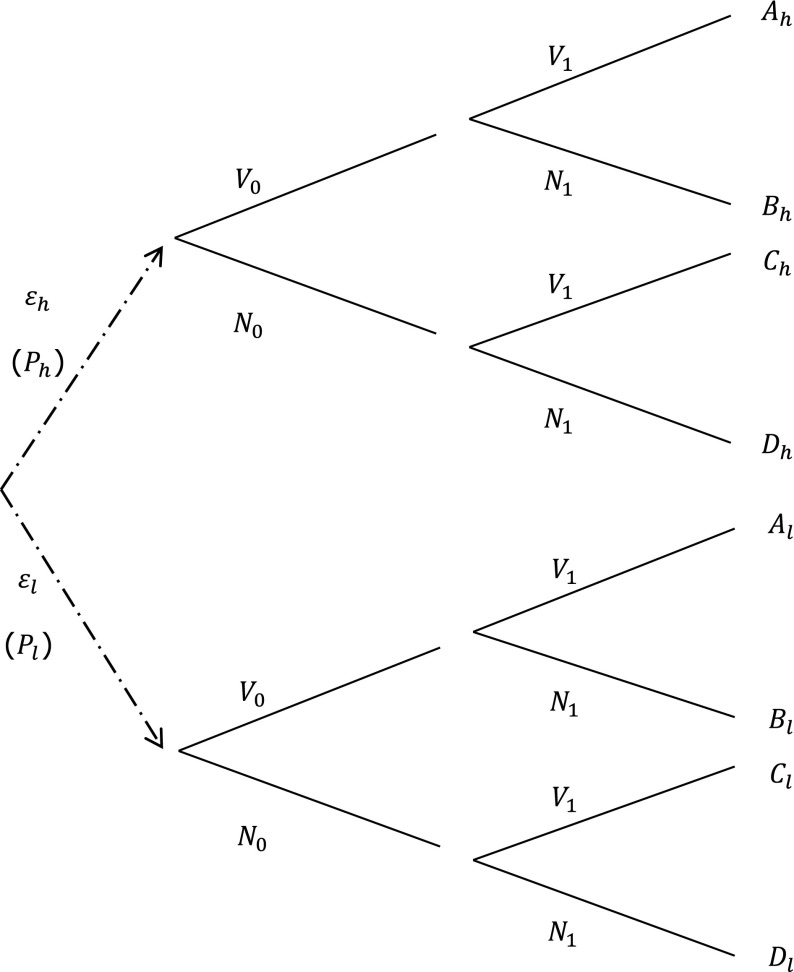
Decision tree for the two-period investment model extended with two possible climate change scenarios (*ε*_*l*_ and *ε*_*h*_)

Climate change impact scenarios can be introduced in the cost-benefit model through defining possible states of nature in order to account for uncertainty about climate change impacts. To illustrate this, consider that under a high climate change impact scenario the annual increase of the flood probability is *ε*_*h*_ and that probability *P*_*h*_ is assigned to this scenario, and that under a low climate change impact scenario, flood probability increases with *ε*_*l*_ with probability *P*_*l*_ = 1 − *P*_*h*_. The corresponding decision tree for this problem is displayed in Fig. 2.

The expected outcome of a strategy is equal to the weighted average of the total discounted costs under the two scenarios. For strategy {*V*_0_; *V*_1_}, the total discounted expected costs are:

3 $$ A={P}_l{A}_l+{P}_h{A}_h, $$

and idem for outcomes *B*, *C* and *D*. Again, the optimal strategy follows from: min{*A*, *B*, *C*, *D*}. In this example, only two climate scenarios are considered. The general case would require a continuous prior distribution of climate change impacts.

The importance of probability distributions for climate change adaptation has, for example, been explained in the United Kingdom Climate Projections 2009 (UKCP09) projections, in which probabilistic climate projections of over-land changes have been derived for the UK (Murphy et al. 2009). However, probabilistic coastal projections are generally not available (Lowe et al. 2009; IPCC 2014). Without probabilistic impact projections, the above method can only employ probabilistic assumptions to derive expected flood damages under climate change for different flood risk management strategies. As a consequence, cost-benefit solutions may appear to be precise, while the discretised probabilities or densities to arrive at the solutions are not (Hall 2007). Risk-based cost-benefit optimisation using probability-weighted scenarios or distributions, therefore, cannot provide final answers to optimal flood risk management decisions. However, it supports the identification of economically efficient management strategies using information that is available to the best of our knowledge. Yet, this information is debatable. Sensitivity analysis can provide insights in the sensitivity of solutions to distributional assumptions.

### Models of learning

#### Modelling of scientific progress

**Fig. 3:**
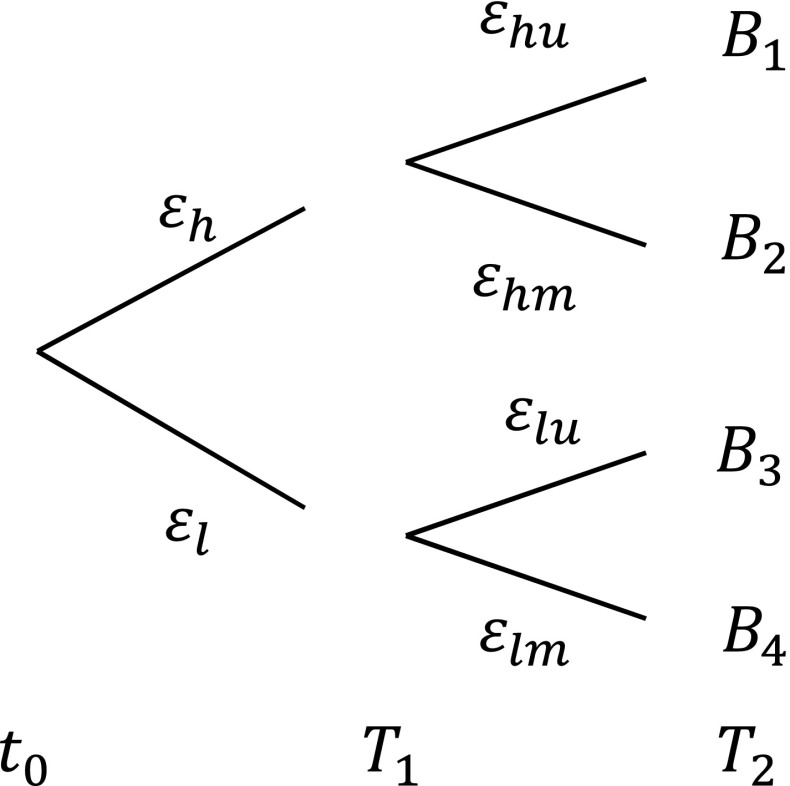
Graphical representation of gradual uncertainty reduction. Outcomes for strategy {*V*_0_; *N*_1_} are displayed

Scientific progress may eventually lead to a better understanding of the severity of climate change. This can be modelled as a probabilistic reduction of climate change uncertainty over time. Figure 3 displays a simplified example of the gradual reduction of sea level rise uncertainty over time. At *t*_0_, sea level rise can be high or low; at *T*_1_, the rate of sea level rise is approximately known; and at *T*_2_, sea level rise uncertainty is fully resolved, and we know the outcome with certainty, for example, *B*_1_.

The initial investment for this setting follows from:

4 $$ \underset{\left\{{V}_0;{V}_1\right\},\left\{{V}_0;{N}_1\right\},\left\{{N}_0;{V}_1\right\},\left\{{N}_0;{N}_1\right\}}{ \min}\left\{{\displaystyle \sum_j}{P}_j\left({\varepsilon}_j\right){A}_j,{\displaystyle \sum_j}{P}_j\left({\varepsilon}_j\right){B}_j,{\displaystyle \sum_j}{P}_j\left({\varepsilon}_j\right){C}_j,{\displaystyle \sum_j}{P}_j\left({\varepsilon}_j\right){D}_j,\right\}, $$

and the investment at decision moment *t*_1_ depends on the available information (*ε*_*l*_ or *ε*_*h*_) at this moment. Larger problems with uncertainty reduction can be formulated recursively by dynamic programming, for example, applied in van der Pol et al. (2014). Clearly, future information has expected value. As a consequence, investment decisions may be changed by the probability of future information arrival, which has a general tendency to reduce overall investment before the arrival of new information, for example, by postponing investment (deferral), changing the scale of investment (e.g. contraction), adaptive design or alternative portfolio choices (switching). These are typical examples of real options strategies from the real options literature. However, real option methods have not often been applied to economic flood risk management studies (Schwartz and Trigeorgis 2001; Woodward et al. 2011). This may be explained by the probabilistic assumptions needed regarding information arrival. In Section 2.3.1, we discussed that there is no consensus about the timing of uncertainty reduction, if reduced at all.

#### Belief updating with new observations

Evidence-based learning can be approached from a frequentist or a Bayesian perspective. Frequentist approaches interpret observations, for example, of extreme water levels, as random realisations from a “true” but unknown distribution. Re-sampling methods, such as bootstrapping, can be used to evaluate the robustness of the initial estimates. Climate change, however, introduces a trend in the data which can be studied with, amongst others, moving window analysis or regression methods. In a moving window analysis, a part of the available observations is treated as if it is stationary (De Michele et al. 1998). For every time step, for example, every year, an extreme value distribution *f* is re-estimated with the last *N* years of observations with standard statistical procedures such as maximum likelihood or the method of L moments. The likelihood of distributional estimates can then be studied by simulation of new observations based on climate scenarios.

Contrary to frequentist approaches, Bayesian inference methods assume that the unknown parameter *θ* is a random variable and can be expressed by means of prior beliefs *P*(*θ*). This is a probabilistic specification of the decision-maker’s beliefs before new evidence has been observed. Prior beliefs can be updated with sample data using Bayes law:

5 $$ P\left(\theta \Big|y\right)=\frac{P\left(y\Big|\theta \right)P\left(\theta \right)}{P(y)}, $$

where *P*(*θ*|*y*) is the posterior belief after observing new event *y*, and *P*(*θ*) is the prior belief. *P*(*y*|*θ*) denotes the likelihood function being the conditional probability distribution of observed data. Prior beliefs about, for example, extreme value distribution parameters, can either be based on subjective guesses (Huard et al. 2010) or can make use of existing information. Bayes law allows combining subjective beliefs with evidence gained from observed data, or simulated data *y* that can be added to original data, in order to arrive at posterior beliefs (Rajabalinejad and Demirbilek 2013). The posterior probability distribution characterises beliefs about the hypothesis *θ* (e.g. increase of mean temperature, sea level rise) after seeing the data. An early application of Bayes theorem to identify optimal protection levels for dike design under limited data and flood uncertainty is found in Davis et al. (1972).

As for the case of perfect learning, optimal investment strategies with evidence-based learning can be studied by formulating the general investment problem (Eq. 2) recursively. The transition probabilities can be derived from the belief updating process which follows from dynamic application of Bayes law (Eq. 5), and simulation of future hydro-meteorological observations. Implementation of this setting is, however, complex and goes beyond the scope of this paper. Note that variability in extremes is high relative to the expected changes in frequency of extreme weather events, which suggests that effects from variability in such settings may well be greater than the structural distributional changes due to climate change in the coming decades, and could increase optimal initial investment. So far, to our knowledge, only few flood risk management studies explore the impacts of future weather variability and resulting transitions in beliefs on current investment decisions.

#### Modelling of disasters

In public choice theory, the outcome of the net loss-minimising model (Eq. 2) has been called the social planner’s solution. It has, however, been argued that social planner solutions may not be implemented, because it differs from policymakers’ preferences which are subject to various factors including the discontent of voters (Hansen and Thisse 1981). Voters’ preferences for flood protection may differ from a social planner perspective, as voters may be risk-averse, or at least have a different planning horizon than the social planner.

Consider, as an example, voters who minimise total expenses on taxes and total costs from flood damages during their lifetime. Consider, further, that voters can observe whether or not flood disasters have occurred in previous years, and that the discounted costs associated with a single flood disaster are typically larger than the present value of total flood protection costs during the lifetime of a person. Two general implications from the objective function of individual voters can be deducted. First, the willingness-to-pay (WTP) for flood protection of an individual voter is not constant over time due to the probabilistic arrival of new information about the occurrence of disasters over time. As a result of the objective function, the WTP of an individual voter might decrease over time if no disaster occurs, but it might increase after the occurrence of a flood disaster. Second, a decision-maker cannot be sure about the timing of re-investment in flood protection, which now also depends on the stochastic nature of the occurrence of flood disasters. Early or frequent re-investment in flood protection, furthermore, may be costly due to initial costs of flood protection measures. In this setting, therefore, a risk-neutral decision-maker has to account for the risk-averse preferences of voters.

## Robustness analysis

The probabilistic extensions described in Section 3 require subjective probabilistic information on climate change impacts and the arrival of new information. However, climate change uncertainties have been classified as deep (Kandlikar et al. 2005). Several authors have argued that the optimality criterion should be abandoned in the presence of deep uncertainties, and that research effort should focus on the identification of robust strategies that perform relatively well across a range of possible futures (Lempert et al. 2006; Hall et al. 2012).

Various kinds of robustness analysis have been developed, which appears to be the result of the normative nature of the robustness concept. Robustness analysis of adaptation strategies can be used to identify robust solutions in a narrow sense by employing alternative decision criteria, for example, minimax or minimax regret criteria (Clarke 2008). Other robustness methods analyse the performance of robust solutions under different degrees of uncertainty, for example, info-gap theory (Hine and Hall 2010). Moreover, in recent robustness analysis, such as adaptation pathways, stakeholder participation is used to explore uncertainties, preferences, lock-ins and path-dependencies by qualitative methods (Haasnoot et al. 2013).

Robustness approaches differ fundamentally from risk-based optimisation approaches in at least two respects. First, they reject the assumption of risk-based approaches that probabilistic information can be identified for analysis of management strategies. Second, they reject the assumption that welfare-maximising solutions can be identified, and are instead aimed at identifying solutions that are expected to perform relatively well under worst case or a wide range of scenarios.

## Discussion

The probabilistic extensions of CBA described in this paper can be used to analyse and compare the economic efficiency and flexibility of flood management strategies under climate change. They thereby support economic decision-making on flood risk management strategies. However, the results of a risk-based CBA of flood risk management and other climate adaptation strategies using probability-weighted scenarios have to be interpreted with care. Results can be misleading, as assigned probabilities may misrepresent uncertainty (Hall 2007).

We observe that the underlying scientific debate on whether or not climate change uncertainty should be addressed as subjective risk or deep uncertainty is increasingly polarised. In many papers, authors either motivate a risk-based or a robustness approach (Speijker et al. 2000; Hine and Hall 2010; Woodward et al. 2011; Brekelmans et al. 2012; Haasnoot et al. 2013). Both risk-based and robustness approaches are defensible, and due to differences in assumptions regarding decision-maker preferences and implementation of uncertainty, and we argue that they may provide different but complementary insights.

A standard approach to expected value-based optimisation assumes risk neutrality. The common use of CBA of flood risk management strategies reflects that expected outcomes provide an important decision criterion for flood risk management. However, expected value optimisation is not the only relevant decision criterion. Even if probabilities would be properly defined decision-makers might, at least to some degree, be risk-averse. Risk aversion can be included in a risk-based economic analysis of flood risk management strategies if a certain degree of risk-aversion is assumed (Kuijper and Kallen 2012; Wang et al. 2015). However, given the imprecise probabilities associated with the impacts of climate change on flood frequencies and the high consequences of flooding, also other decision-maker preferences may be applicable, such as uncertainty aversion, loss aversion or regret aversion (Woodward and Bishop 1997; Clarke 2008; Weitzman 2009). These preferences do not fit in a standard cost-benefit framework (Hogarth and Kunreuther 1995; Yager 2004; Clarke 2008).

Both CBA and robustness approaches impose extreme assumptions. In a deterministic CBA uncertainty is largely ignored, while usually risk-neutrality with known probabilities is assumed in an expected value-based CBA (Watkiss et al. 2014). Robustness approaches usually put implicit or explicit weights on possible outcomes. For example, some decision criteria which are employed to obtain robust solutions, such as minimisation of maximum losses or maximum regret, focus only on outcomes under worst case scenarios. Other robust decision criteria employ arbitrary scenario weights, or consider scenario outcomes to be equally likely (Gaspars-Wieloch 2014).

Yet, it may not always be the case that the outcomes from risk-based optimisation and robustness analysis are divergent due to similarities between the overall decision objectives. For example, recently robustness methods and approaches have been developed to study adaptive flood risk management and other climate adaptation strategies that can be changed at relatively low costs over time (Kwadijk et al. 2010; Merz et al. 2010; Haasnoot et al. 2013). This refers to the overall objective of decision robustness, which is important for flood risk management in addition to the robustness of a system to withstand disturbances (Mens et al. 2011). Risk-based optimisation models with events of information arrival also consider decision robustness by quantitative evaluation of the flexibility of management strategies under the emergence of new information.

To understand the quantitative effects of learning presented in this paper, further applied economic analyses of flood risk management strategies is required, with extensions to implement the different types of learning. Sensitivity analysis allows the study of optimal long-run flood protection without the explicit modelling of information arrival (Zhu et al. 2007). However, anticipation of possible future information may help to avoid costly lock-in situations by giving weight to information scenarios in which non-incremental adaptation decisions would be required. Non-incremental changes in adaptation strategies tend to be costly, but early implementation could in some cases reduce long-run adaptation costs (Kates et al. 2012).

## Conclusions

Climate change has introduced additional challenges for the economic analysis of flood risk management strategies. At the local level decisions need to be made on investment in flood risk reduction. At the global level, strategies need to be defined on how to allocate adaptation funds for flood risk management in various regions of the world. This paper has discussed probabilistic extensions of cost-benefit analysis to identify economically efficient strategies under climate change. Uncertainty about the changes in return periods of hydro-meteorological extremes was introduced by probability-weighted scenarios. We revisited expert elicitation as a means to study climate change uncertainty. Expert elicitation is controversial. Yet, there appears to be some consensus that at least some of the more extreme climate scenarios are less likely than others in the near term.

Learning about climate change impacts has remained a largely unexplored domain in flood risk management. In the long run, uncertainty may be reduced because of scientific progress and longer time series of hydro-meteorological observations. Uncertainty reduction from statistical analysis of hydro-meteorological observations is, however, not very likely in the near term as trend detection tests may remain inconclusive in the coming decades, and convergence will be slow due to the high degree of variability in these observations. We have discussed that investment responses in the past have been strongly driven by actual observations, both to evaluate the performance of water systems, for example, with a calibrated rainfall generator, and by the occurrence of disasters. The analysis of the likelihood of and investment responses to possible climate change signals, either as a result of transitions in beliefs about climate change impacts, or induced by incidents, may therefore improve the economic efficiency of decisions on flood risk management strategies. We have argued that risk-based approaches reflect different decision-maker preferences and implementations of climate uncertainty than robustness approaches. We have highlighted that flood risk practitioners and policymakers are not merely concerned with subjective estimates of expected outcomes. We therefore advocate the use of robustness methods in addition to, or combined with, cost-benefit analysis for the economic analysis of flood risk management strategies to support decisions. For further research, it would be interesting to combine cost-benefit and robustness solutions in a meta-analysis.

Our paper contributes to the development of adaptation strategies through economic analysis of flood risk reduction under climate change. Local investments can be optimised, and global strategies on the allocation of adaptation funds can be enhanced if the costs and benefits of flood risk management strategies of individual countries are understood. Global strategies on the allocation of adaptation funds for flood risk management can then be derived from an economic analysis of costs and benefits of flood risk reduction that considers uncertainty under climate change.

## References

[CR1] Andronova NG, Schlesinger ME (2001). Objective estimation of the probability density function for climate sensitivity. J Geophys Res.

[CR2] Arnbjerg-Nielsen K (2012). Quantification of climate change effects on extreme precipitation used for high resolution hydrologic design. Urban Water J.

[CR3] Arnell NW, Tompkins EL, Adger WN (2005). Eliciting information from experts on the likelihood of rapid climate change. Risk Anal.

[CR4] Bao Y, Tung Y-K, Hasfurther VR (1987). Evaluation of uncertainty in flood magnitude estimator on annual expected damage costs of hydraulic structures. Water Resour Res.

[CR5] Berggren K, Packman J, Ashley R, Viklander M (2014) Climate changed rainfalls for urban drainage capacity assessment. Urban Water J 11(7):543–556

[CR6] Boardman AE, Greenberg DH, Vining AR, Weimer DL (2011). Cost-benefit analysis: concepts and practice.

[CR7] Boughton W, Droop O (2003). Continuous simulation for design flood estimation—a review. Environ Model Softw.

[CR8] Bouwer LM, Bubeck P, Aerts JCJH (2010). Changes in future flood risk due to climate and development in a Dutch polder area. Global Environ Chang.

[CR9] Brekelmans R, den Hertog D, Roos K, Eijgenraam C (2012). Safe dike heights at minimal costs: the nonhomogeneous case. Oper Res.

[CR10] Cameron D (2006). An application of the UKCIP02 climate change scenarios to flood estimation by continuous simulation for a gauged catchment in the northeast of Scotland, UK (with uncertainty). J Hydrol.

[CR11] Cameron DS, Beven KJ, Tawn J, Blazkova S, Naden P (1999). Flood frequency estimation by continuous simulation for a gauged upland catchment (with uncertainty). J Hydrol.

[CR12] Clarke H (2008). Classical decision rules and adaptation to climate change. Aust J Agr Resour Ec.

[CR13] Coles S, Pericchi L (2003). Anticipating catastrophes through extreme value modelling. J Roy Stat Soc C-App.

[CR14] Copeland T, Antikarov V (2003). Real options: a practitioner’s guide.

[CR15] Dalkey N, Helmer O (1963). An experimental application of the Delphi method to the use of experts. Manag Sci.

[CR16] Davis DR, Kisiel CC, Duckstein L (1972). Bayesian decision theory applied to design in hydrology. Water Resour Res.

[CR17] de Bruin K, Ansink E (2011). Investment in flood protection measures under climate change uncertainty. Clim Change Econ.

[CR18] De Michele C, Montanari A, Rosso R (1998). The effects of non-stationarity on the evaluation of critical design storms. Water Sci Technol.

[CR19] de Moel H, Aerts JCJH (2011). Effect of uncertainty in land use, damage models and inundation depth on flood damage estimates. Nat Hazards.

[CR20] Dierauer J, Pinter N, Remo JWF (2012). Evaluation of levee setbacks for flood-loss reduction, Middle Mississippi River, USA. J Hydrol.

[CR21] Fowler HJ, Wilby RL (2010). Detecting changes in seasonal precipitation extremes using regional climate model projections: implications for managing fluvial flood risk. Water Resour Res.

[CR22] Gaspars-Wieloch H (2014) Modifications of the Hurwicz’s decision rule. Central Europ J Operations Res 22:779–794

[CR23] Gerritsen H (2005). What happened in 1953? The big flood in the Netherlands in retrospect. Philos Transact Math Phys Eng Sci.

[CR24] Gersonius B, Ashley R, Pathirana A, Zevenbergen C (2013). Climate change uncertainty: building flexibility into water and flood risk infrastructure. Clim Chang.

[CR25] Gollier C, Jullien B, Treich N (2000). Scientific progress and irreversibility: an economic interpretation of the ‘precautionary principle’. J Public Econ.

[CR26] Groisman PY, Knight RW, Easterling DR, Karl TR, Hegerl GC, Razuvaev VN (2005). Trends in intense precipitation in the climate record. J Clim.

[CR27] Haasnoot M, Kwakkel JH, Walker WE, ter Maat J (2013). Dynamic adaptive policy pathways: a method for crafting robust decisions for a deeply uncertain world. Global Environ Chang.

[CR28] Hall J (2007). Probabilistic climate scenarios may misrepresent uncertainty and lead to bad adaptation decisions. Hydrol Process.

[CR29] Hall J, Sayers P, Dawson R (2005). National-scale assessment of current and future flood risk in England and Wales. Nat Hazards.

[CR30] Hall JW, Lempert RJ, Keller K, Hackbarth A, Mijere C, McInerney DJ (2012). Robust climate policies under uncertainty: a comparison of robust decision making and info-gap methods. Risk Anal.

[CR31] Hallegatte S, Shah A, Lempert R, Brown C, Gill S (2012) Investment decision making under deep uncertainty: application to climate change. World Bank, Working paper, WPS6193

[CR32] Hamed KH (2008). Trend detection in hydrologic data: the Mann–Kendall trend test under the scaling hypothesis. J Hydrol.

[CR33] Hanley ME, Hoggart SPG, Simmonds DJ, Bichot A, Colangelo MA, Bozzeda F, Heurtefeux H, Ondiviela B (2014). Shifting sands? Coastal protection by sand banks, beaches and dunes. Coast Eng.

[CR34] Hansen P, Thisse J-F (1981). Outcomes of voting and planning: Condorcet, Weber and Rawls locations. J Public Econ.

[CR35] Harries T, Penning-Rowsell E (2011). Victim pressure, institutional inertia and climate change adaptation: the case of flood risk. Global Environ Chang.

[CR36] Hay LE, Wilby RL, Leavesley GH (2000). A comparison of delta change and downscaled GCM scenarios for three mountainous basins in the United States. J Am Water Resour As.

[CR37] Hine D, Hall JW (2010). Information gap analysis of flood model uncertainties and regional frequency analysis. Water Resour Res.

[CR38] Hoes O, Schuurmans W (2006). Flood standards or risk analyses for polder management in the Netherlands. Irrig Drain.

[CR39] Hogarth R, Kunreuther H (1995). Decision making under ignorance: arguing with yourself. J Risk Uncertain.

[CR40] Hooijer A, Klijn F, Pedroli GBM, Van Os AG (2004). Towards sustainable flood risk management in the Rhine and Meuse River basins: synopsis of the findings of IRMA-SPONGE. River Res Applic.

[CR41] Huard D, Mailhot A, Duchesne S (2010). Bayesian estimation of intensity–duration–frequency curves and of the return period associated to a given rainfall event. Stoch Environ Res Risk Assess.

[CR42] Ingham A, Ma J, Ulph A (2007). Climate change, mitigation and adaptation with uncertainty and learning. Energ Policy.

[CR43] Field CB, Barros VR, Dokken DJ, Mach KJ, Mastrandrea MD, Bilir TE, Chatterjee M, Ebi KL, Estrada YO, Genova RC, Girma B, Kissel ES, Levy AN, MacCracken S, Mastrandrea PR, White LL, IPCC (2014). Climate Change 2014: impacts, adaptation, and vulnerability. Part A: Global and sectoral aspects. Contribution of Working Group II to the Fifth Assessment Report of the Intergovernmental Panel on Climate Change.

[CR44] Jongman B, Ward PJ, Aerts JCJH (2012). Global exposure to river and coastal flooding: long term trends and changes. Global Environ Chang.

[CR45] Jonkman SN, Bočkarjova M, Kok M, Bernardini P (2008). Integrated hydrodynamic and economic modelling of flood damage in the Netherlands. Ecol Econ.

[CR46] Kandlikar M, Risbey J, Dessai S (2005). Representing and communicating deep uncertainty in climate-change assessments. C R Geosci.

[CR47] Kates RW, Travis WR, Wilbanks TJ (2012). Transformational adaptation when incremental adaptations to climate change are insufficient. Proc Natl Acad Sci.

[CR48] Katsman CA, Sterl A, Beersma JJ, Brink HW, Church JA, Hazeleger W, Kopp RE, Kroon D (2011). Exploring high-end scenarios for local sea level rise to develop flood protection strategies for a low-lying delta—the Netherlands as an example. Clim Chang.

[CR49] Khaliq MN, Ouarda TBMJ, Ondo JC, Gachon P, Bobée B (2006). Frequency analysis of a sequence of dependent and/or non-stationary hydro-meteorological observations: a review. J Hydrol.

[CR50] Kind JM (2014). Economically efficient flood protection standards for the Netherlands. J Flood Risk Manage.

[CR51] Knutti R, Furrer R, Tebaldi C, Cermak J, Meehl GA (2010). Challenges in combining projections from multiple climate models. J Clim.

[CR52] Kolstad CD (1994). George Bush versus Al Gore: irreversibilities in greenhouse gas accumulation and emission control investment. Energ Policy.

[CR53] Kron W (2005). Flood risk = hazard • values • vulnerability. Water Int.

[CR54] Kuijper B, Kallen MJ (2012). Uncertainty in optimal decisions for dike maintenance. Struct Infrastr Eng.

[CR55] Kundzewicz Z, Hirabayashi Y, Kanae S (2010). River floods in the changing climate—observations and projections. Water Resour Manag.

[CR56] Kwadijk JCJ, Haasnoot M, Mulder JPM, Hoogvliet MMC, Jeuken ABM, van der Krogt RAA, van Oostrom NGC, Schelfhout HA (2010). Using adaptation tipping points to prepare for climate change and sea level rise: a case study in the Netherlands. WIREs Clim Chg.

[CR57] Leach AJ (2007). The climate change learning curve. J Econ Dyn Contr.

[CR58] Lempert RJ, Groves DG, Popper SW, Bankes SC (2006). A general, analytic method for generating robust strategies and narrative scenarios. Manag Sci.

[CR59] Levy B, McCuen R (1999). Assessment of storm duration for hydrologic design. J Hydrol Eng.

[CR60] Lickley MJ, Lin N, Jacoby HD (2015) Analysis of coastal protection under rising flood risk. Climate Risk Manage, http://dx.doi.org/10.1016/j.crm.2015.01.001

[CR61] Lowe JA, Howard TP, Pardaens A, Tinker J, Holt J, Wakelin S, G. M, Leake J et al (2009) UK Climate Projections science report: Marine and coastal projections. Met Office Hadley Centre, Exeter, UK

[CR62] Mailhot A, Lachance-Cloutier S, Talbot G, Favre A-C (2013). Regional estimates of intense rainfall based on the peak-over-threshold (POT) approach. J Hydrol.

[CR63] Mays LW (2011). Water resources engineering.

[CR64] Mens MJP, Klijn F, de Bruijn KM, van Beek E (2011). The meaning of system robustness for flood risk management. Environ Sci Pol.

[CR65] Merz B, Thieken AH (2005). Separating natural and epistemic uncertainty in flood frequency analysis. J Hydrol.

[CR66] Merz B, Hall J, Disse M, Schumann A (2010). Fluvial flood risk management in a changing world. Nat Hazards Earth Syst Sci.

[CR67] Meyer V, Priest S, Kuhlicke C (2012). Economic evaluation of structural and non-structural flood risk management measures: examples from the Mulde River. Nat Hazards.

[CR68] Milly PCD, Wetherald RT, Dunne KA, Delworth TL (2002). Increasing risk of great floods in a changing climate. Nature.

[CR69] Milly PCD, Betancourt J, Falkenmark M, Hirsch RM, Kundzewicz ZW, Lettenmaier DP, Stouffer RJ (2008). Stationarity is dead: whither water management?. Science.

[CR70] Murphy JM, Sexton DMH, Jenkins GJ, Boorman PM, Booth BBB, Brown CC, Clark RT, Collins M (2009). UK Climate Projections Science Report: Climate change projections.

[CR71] Narain U, Margulis S, Essam T (2011). Estimating costs of adaptation to climate change. Clim Pol.

[CR72] NBW (2005) Het Nationaal Bestuursakkoord Water, Available at: http://www.helpdeskwater.nl/onderwerpen/wetgeving-beleid/nationaal/@1280/nationaal/ [‘Dutch Governance Agreement Water’, in Dutch]

[CR73] Neumayer E, Plümper T, Barthel F (2014). The political economy of natural disaster damage. Global Environ Chang.

[CR74] Nilsen V, Lier JA, Bjerkholt JT, Lindholm OG (2011). Analysing urban floods and combined sewer overflows in a changing climate. J Water Clim Change.

[CR75] Pathirana A, Tsegaye S, Gersonius B, Vairavamoorthy K (2011). A simple 2-D inundation model for incorporating flood damage in urban drainage planning. Hydrol Earth Syst Sci.

[CR76] Press SJ (2003). Subjective and objective Bayesian statistics: principles, models, and applications.

[CR77] Purvis MJ, Bates PD, Hayes CM (2008). A probabilistic methodology to estimate future coastal flood risk due to sea level rise. Coast Eng.

[CR78] Rajabalinejad M, Demirbilek Z (2013). A Bayesian probabilistic approach for impacts of sea level rise on coastal engineering design practice. Ocean Eng.

[CR79] Roe GH, Baker MB (2007). Why is climate sensitivity so unpredictable?. Science.

[CR80] Schwartz ES, Trigeorgis L (2001). Real options and investment under uncertainty: classical readings and recent contributions.

[CR81] Smith JB (1997). Setting priorities for adapting to climate change. Global Environ Chang.

[CR82] Speijker LJ, van Noortwijk JM, Kok M, Cooke RM (2000). Optimal maintenance decisions for dikes. Probab Eng Inf Sci.

[CR83] Stainforth DA, Aina T, Christensen C, Collins M, Faull N, Frame DJ, Kettleborough JA, Knight S (2005). Uncertainty in predictions of the climate response to rising levels of greenhouse gases. Nature.

[CR84] Stive MJF, de Schipper MA, Luijendijk AP, Aarninkhof SGJ, van Gelder-Maas C, van Thiel de Vries JSM, de Vries S, de Henriquez M (2013). A new alternative to saving our beaches from sea-level rise: the sand engine. J Coastal Res.

[CR85] van Dantzig D (1956). Economic decision-problems for flood prevention. Econometrica.

[CR86] van der Pol TD, van Ierland EC, Weikard HP (2014). Optimal dike investments under uncertainty and learning about increasing water levels. J Flood Risk Manag.

[CR87] Vaughan DG (2008) West Antarctic Ice Sheet collapse – the fall and rise of a paradigm. Clim Change 91(1–2):65–79

[CR88] Vaughan DG, Spouge JR (2002). Risk estimation of collapse of the West Antarctic ice sheet. Clim Chang.

[CR89] Vrijling JK (2001). Probabilistic design of water defense systems in The Netherlands. Reliab Eng Syst Safe.

[CR90] Wahl T, Haigh ID, Woodworth PL, Albrecht F, Dillingh D, Jensen J, Nicholls RJ, Weisse R (2013). Observed mean sea level changes around the North Sea coastline from 1800 to present. Earth-Sci Rev.

[CR91] Wang L, van Gelder PHAJM, Vrijling JK, Maskey S, Ranasinghe R (2015). Risk-averse economic optimization in the adaptation of river dikes to climate change. Water Resour Manag.

[CR92] Waters D, Watt WE, Marsalek J, Anderson BC (2003). Adaptation of a storm drainage system to accommodate increased rainfall resulting from climate change. J Environ Plan Manag.

[CR93] Watkiss P, Hunt A, Blyth W, Dyszynski J (2014) The use of new economic decision support tools for adaptation assessment: a review of methods and applications, towards guidance on applicability. Climatic Change. doi:10.1007/s10584-014-1250-9

[CR94] Weitzman ML (2009). On modeling and interpreting the economics of catastrophic climate change. Rev Econ Stat.

[CR95] Willems P (2013). Revision of urban drainage design rules after assessment of climate change impacts on precipitation extremes at Uccle, Belgium. J Hydrol.

[CR96] Woodward RT, Bishop RC (1997). How to decide when experts disagree: uncertainty-based choice rules in environmental policy. Land Econ.

[CR97] Woodward M, Gouldby B, Kapelan Z, Khu ST, Townend I (2011). Real options in flood risk management decision making. J Flood Risk Manag.

[CR98] Wu X, Yu D, Chen Z, Wilby R (2012). An evaluation of the impacts of land surface modification, storm sewer development, and rainfall variation on waterlogging risk in Shanghai. Nat Hazards.

[CR99] Yager RR (2004). Decision making using minimization of regret. Int J Approx Reason.

[CR100] Zhang X, Zwiers FW, Li G (2004). Monte Carlo experiments on the detection of trends in extreme values. J Clim.

[CR101] Zhu T, Lund J (2009). Up or out? Economic-engineering theory of flood levee height and setback. J Water Res Pl-ASCE.

[CR102] Zhu T, Lund JR, Jenkins MW, Marques GF, Ritzema RS (2007). Climate change, urbanization, and optimal long-term floodplain protection. Water Resour Res.

[CR103] Zickfeld K, Levermann A, Morgan MG, Kuhlbrodt T, Rahmstorf S, Keith D (2007). Expert judgements on the response of the Atlantic meridional overturning circulation to climate change. Clim Chang.

[CR104] Zoppou C (2001). Review of urban storm water models. Environ Model Softw.

